# Evolving Paradigm in Radioactive Iodine Therapy for Differentiated Thyroid Cancer: Historical Perspectives, Current Practices and Future Directions

**DOI:** 10.3390/diagnostics15111438

**Published:** 2025-06-05

**Authors:** Jasna Mihailović

**Affiliations:** 1 Department of Nuclear Medicine, Faculty of Medicine, University of Novi Sad, Hajduk Veljkova 3, 21000 Novi Sad, Serbia; jasna.mihailovic@mf.uns.ac.rs; 2 Division of Nuclear Medicine, Oncology Institute of Vojvodina, Put dr Goldmana 4, 21204 Sremska Kamenica, Serbia

**Keywords:** thyroid cancer, risk stratification, radioiodine therapy, indications, molecular theranostics

## Abstract

Therapy with radioactive iodine (I-131) following a total thyroidectomy has been a gold standard in the treatment of differentiated thyroid cancer (DTC) for over 80 years. Over the years, its role has shifted from routine use to a more selective, risk-adapted approach, informed by tumor biology, patient risk stratification and evolving clinical guidelines. This review examines the changing landscape of I-131 therapy, tracing its historical foundations, current indications, and future directions shaped by molecular medicine. We discuss the transition from a standardized, one-size-fits-all treatment approach to an individualized, dynamic stratification model that allows for ongoing risk reassessment and tailored treatment strategies. Key updates in clinical practice, such as the 2015 ATA Guidelines, the 2022 ETA Consensus Statement, and joint SNMMI and EANM nuclear medicine recommendations, are critically examined. We also address ongoing controversies in the management of low- and intermediate-risk patients, including the roles of I-131 whole-body scanning, activity selection, and overall treatment approach. Molecular theranostics is ushering in a new era in DTC management, enabling improved patient selection and more precise treatment. Advances in molecular profiling, imaging, and targeted therapies support a personalized treatment approach that aims to optimize therapeutic decisions while minimizing side effects and enhancing long-term safety.

## 1. Introduction

Thyroid neoplasm is the most frequent endocrine malignancy. In recent years, the incidence of thyroid cancer has been rising. According to global cancer statistics, thyroid cancer was the seventh most common malignancy worldwide in 2022 [1]. However, in some countries, including the United States, recent trends have shifted. While thyroid cancer mortality in the U.S. continues to increase, the incidence of new cases has declined by 2% per year since 2014. This decline is largely attributed to changes in clinical practice, including updated thyroid cancer guidelines that discourage screening and the adoption of more restrictive diagnostic criteria with an aim to minimize overdiagnosis [2,3].

## 2. Risk Stratification

Patients diagnosed with differentiated thyroid cancer (DTC) usually show a favorable long-term prognosis with a reported survival rate of more than 95% after a 10-year follow-up. Specifically, the overall survival rates of approximately 93%, 85%, 70%, and 63% were detected after 5, 10, 20 and 30 years of initial diagnosis, respectively [4,5]. The recurrence rate in DTC varies widely, ranging from 4% to 42%, depending on tumor histology, follow-up period, and risk stratification [6,7]. In regard to risk stratification, recurrence develops in less than 3–13% of low-risk patients, in 21–36% of intermediate-risk patients, and approximately up to 68% of high-risk patients [8]. In a recent study, 75 out of 685 patients with well-differentiated thyroid cancer (WDTC), representing 11%, experienced a structural recurrence in the cervical region. Additionally, 36 patients (5.2%) developed distant metastases, with 2.8% showing both local and distant disease [9].

Despite the relatively low mortality rate associated with DTC, disease recurrence remains a significant clinical concern during long-term follow-up. Several risk factors influence patient outcomes, including age, tumor size, tumor histology, extrathyroidal extension, and the presence of regional lymph nodes or distant metastases. Staging (i.e., prognostic) systems apply statistical models to combined risk factors to stratify patients into low- or high-risk categories for recurrence or disease-related mortality. Staging systems have a role in assessing individual patient prognosis, guiding postoperative therapeutic decisions, estimating the risk of recurrence of disease and disease-specific mortality, and planning the appropriate frequency of follow-up. In addition, the standardized risk stratification system for DTC patients improves communication among healthcare professionals, facilitates medical database searches, and supports the development of clinical studies and trials [10].

Over time, various staging classification systems have been established by different authors and centers worldwide. The most commonly used and cited staging systems include the EORTC, AMES, AGES, MACIS, MSK, and TNM systems. The EORTC system was the first staging system for thyroid cancer, developed in 1979, that was based on age, gender, histology, degree of tumor differentiation, tumor invasion, and distant metastases [11]. The AMES system, introduced in 1988 at the Lahey Clinic, incorporates age, presence of distant metastases, tumor extension, and tumor size [12]. The AGES system, established at the Mayo Clinic, utilizes age, tumor grade (Broder’s classification), tumor extension (including local invasion and distant metastases), and tumor size [13]. The MACIS system, developed in 1993, was an update of the AGES system, which was introduced at the Mayo Clinic in 1987. This prognostic system applies only to papillary thyroid carcinoma (PTC) and includes metastases (distant metastases), age, completeness of surgery, tumor invasion, and size [14]. The MSK-GAMES was established at Memorial Sloan Kettering Cancer Center and published in 1994. It was based on grade, age, metastases, extrathyroidal extension, size, and histology (tumor type and differentiation) [15]. The TNM classification of malignant tumors (TNM) was established by the American Joint Committee on Cancer (AJCC) and the Union Internationale Contre le Cancer (UICC). The TNM staging system was based on several characteristics, including tumor size (T), lymph node spread (N), and metastatic spread (M). This classification system can be either clinical (cTNM), based on pre-treatment medical evidence, or pathological (pTNM), based on surgical and histopathological data. It evaluates three key characteristics: tumor size (T), presence (N1) or absence (N0) of metastatic lymph nodes, and presence (M1) or absence (M0) of distal metastases [16,17,18]. It was developed in 1987, revised in 1992, and finalized for publication in 1997. Over the years, updated versions were published in 2002, 2010, and 2018, corresponding to the 6th, 7th, and 8th editions, respectively (Table 1 and Table 2).

While systems like TNM, AMES, and EORTC primarily identify risk factors associated with overall mortality/survival, others—such as TNM, MACIS, AGES, and MSK-GAMES focus on predicting disease-specific mortality [10].

Despite efforts to establish a standardized approach, no single staging system for DTC has gained universal acceptance. A comparative study assessing the prognostic accuracy of the TNM, AGES, MACIS, and AMES systems found no statistically significant differences in predictive performance, providing no definitive evidence to support one model over the others. Nevertheless, several leading organizations—including the AJCC, the American Cancer Society (ACS), the National Comprehensive Cancer Network (NCCN), and the American College of Surgeons—have endorsed the TNM classification as the international standard for staging DTC patients [19].

Although the TNM system predicts the risk of disease-specific mortality, it is not well-suited for accurately assessing the risk of recurrence or persistent disease in DTC. Since the recurrence risk in DTC patients is higher than the mortality risk, The American Thyroid Association (ATA) 2009 guidelines recommended a new risk stratification system. This system classifies DTC patients into three categories, including low, intermediate, and high risk for recurrence and persistent disease [20].

Besides prognostic risk factors for disease/specific mortality and recurrence, initial risk stratification should also include surgical reports, histopathological reports, postoperative ultrasound, and serum thyroglobulin (Tg) values [21].

Initial risk stratification guides the early therapeutic algorithm and determines whether patients should receive postoperative radioiodine therapy. However, during the follow-up, a patient’s risk for recurrence may change based on their response to the applied treatment [22]. In 2010, Tuttle et al. were the first to propose a risk-adapted approach that refined the ATA risk estimates during the first two years of patient monitoring following total thyroidectomy and radioiodine therapy. By incorporating imaging findings, Tg levels, and response to initial treatment, they demonstrated that this ongoing—i.e., dynamic risk stratification, more accurately predicts long-term clinical outcomes in DTC patients [23].

## 3. Historical Overview

### 3.1. Beginnings of Radioactive Iodine-131Administration in DTC

The use of radioactive iodine therapy began in the United States with Dr. Saul Hertz at Massachusetts General Hospital. He is considered the father of theranostics for pioneering the concept through the clinical application of radioactive iodine. In the early 1930s, Hertz believed that radioactive iodine “will be a useful therapy method in cases of over-activity of the thyroid gland” [24]. Soon after, in 1937, he and physicists from the Massachusetts Institute of Technology began experimental research on iodine uptake in rabbit thyroids using I-128 and presented their results [25]. In March 1941, Hertz and Roberts applied the first targeted radionuclide treatment. They performed multiple courses of radioactive iodine [a mixture of the radioactive iodine-130 (I-130) and radioactive iodine-131 (I-131)] on hyperthyroid patients [26].

Around the same time, in New York, radioiodine treatment was administered for thyroid cancer at two institutions. The first administration was carried out by Dr. Albert Keston and his colleagues at Columbia University. They treated a thyroid cancer patient with radioiodine following a subtotal thyroidectomy and reported increased uptake in bone metastases [27]. Dr. Samuel Seidlin, at Montefiore Hospital in New York, administered I-131 for thyroid cancer on May 11, 1943. He treated hyperthyroidism secondary to extensive functioning metastases in patients who had previously undergone thyroidectomy. During the following 22 months, the patients received both I-130 and I-131 in cumulative activity amounts of 9.94 GBq (i.e., 268.8 mCi) [28]. After several years, in 1948, Seidlin concluded that eliminating the thyroid remnants is necessary for optimal radioiodine uptake by thyroid cancer metastases [29].

Meanwhile, on the West Coast, Blahd and his team at UCLA routinely treated patients with metastatic DTC using I-131, while on the East Coast, Beierwaltes studied the efficacy of radioiodine treatment. He advocated that the primary role of radioactive iodine (RAI) is to destroy postoperative thyroid remnants, thereby facilitating patient follow-up [30].

### 3.2. Beginnings of Radioiodine Ablation of DTC

Back in the 70s, Beierwaltes’ research team at the University of Michigan was the first to suggest that postoperative radioiodine therapy could reduce the mortality rate in DTC patients aged 40 and older. They reported a 20-fold mortality reduction in patients treated with I-131 versus those who were treated by surgery alone [31]. Similarly, Krishnamurthy and Blahd reported a positive impact of radioiodine therapy on patient survival. In a 25-year prospective study involving 54 patients with thyroid cancer of various histological types, they determined that I-131 ablation was the most influential factor in survival outcomes [32]. Several years later, Beierwaltes established a three-step postoperative algorithm: the first step involved total thyroidectomy; the second required thyroid hormone withdrawal for 4–6 weeks; and the third consisted of a whole-body scan to detect postoperative thyroid remnants and metastases. He advocated tailoring radioiodine therapy based on the site of uptake, recommending 5.55 GBq (i.e., 150 mCi) to ablate the thyroid remnant, 6.475 GBq (i.e., 175 mCi) for regional node metastases, and 7.4 GBq (i.e., 200 mCi) for distant metastases outside the neck. Through this approach, he established the rationale for radioiodine treatment in DTC patients [33,34]. Shortly thereafter, post-surgical remnant ablation with radioiodine became routine in DTC management. Radioactive iodine was routinely administered to all DTC patients, optimally 4–6 weeks after near-total or total thyroidectomy.

During the forthcoming years, Mazzaferri and his team extensively studied the impact of I-131 on patient outcomes [35,36,37,38]. In 1977, they found a notable difference in recurrence and mortality between patients who received I-131 treatment and those treated with other therapeutic options (for recurrence, *p* < 0.001; for mortality, *p* < 0.01) [35]. The same authors also suggested that I-131 effectively reduces recurrence and mortality rates in patients over 40 years old with tumors larger than 1.5 cm, bilateral disease, metastases, or local invasion [36]. After updating their previous results, Mazzaferi et al. observed a reduced recurrence rate across all age groups and a decrease in mortality rate in patients ≥ 40 years at the time of diagnosis. In contrast, no benefit was seen in patients with unifocal tumors smaller than 1.5 cm in size, without metastatic lymph nodes or thyroid capsule invasion [37]. In a study with a 30-year follow-up, Mazzaferri et al. reported a 16% recurrence in patients treated with RAI (ablative or adjuvant therapy) compared to 38% of those treated with thyroxine alone. Additionally, cause-specific mortality was significantly lower in patients treated with radioiodine (3%) compared to those who did not undergo radioiodine therapy (8%) [38].

On the contrary, researchers from the Mayo Clinic had differing views on the efficacy of remnant ablation. Sisson argued that the current literature data does not support the notion that remnant ablation decreases recurrence in DTC patients [39]. Similarly, Snyder et al. stated that “successful ablation does not prevent recurrence of tumor” [40]. Shortly after the introduction of the MACIS system for predicting mortality in PTC patients at the Mayo Clinic, Hay and colleagues revised the standard approach, advocating for the selective use of remnant ablation. They found that routine ablation for low-risk patients did not significantly improve outcomes. This selective approach recommended omitting ablation in low-risk patients [41,42,43,44,45]. In a more recent study conducted on 2952 low-risk adult PTC patients (MACIS scores > 6) treated from 1953 to 2014, the researchers concluded that radioactive iodine remnant ablation did not reduce mortality or recurrence [46].

### 3.3. Radioiodine Activity Selection

Shortly after the introduction of radioiodine treatment into the routine postoperative management of patients with DTC, high-activity radioiodine ablation became a standard clinical practice for many years. However, the optimal activity of I-131 for effective ablation remains a subject of debate. While some researchers advocate for high activity levels, others recommend lower activity doses. Consequently, the administered I-131 activity has varied between 1.1 GBq (i.e., 30 mCi) and 3.7 GBq (i.e., 100 mCi). In a meta-analysis, Doi et al. demonstrated that 1.1 GBq (i.e., 30 mCi) of I-131 was less effective in ablating thyroid remnants compared to higher doses, ranging from 2.8 (i.e., ~75.67 mCi) to 3.7 GBq (i.e., 100 mCi) [47]. Kukulska et al. reported that a 1.1 GBq dose of radioiodine was less effective than a 2.22 GBq (i.e., 60 mCi) dose [48]. Moreover, they found that the therapeutic efficacy of a 2.22 GBq (i.e., 60 mCi) dose was comparable to that of a 3.7 GBq dose (i.e., 100 mCi). Prpic et al. evaluated 259 patients with DTC confined to the thyroid and found that 3.7 GBq (i.e., 100 mCi) of I-131 was significantly more effective in thyroid ablation than doses ranging from 1.1 GBq to 1.85 GBq (i.e., 50 mCi), and superior to a 2.775 GBq dose (i.e., 75 mCi) [49]. Furthermore, Pacini et al. emphasized that when recombinant human thyroid-stimulating hormone (rhTSH) stimulation is used, a standard dose of 1.1 GBq (i.e., 30 mCi) of I-131 is insufficient for achieving effective thyroid ablation [50].

In contrast, the concept of low-dose iodine-131 remnant ablation was introduced in the late 1970s, and since then, its efficacy has been confirmed by numerous centers. For many years, a large number of North American institutions have routinely administered an I-131 activity of 1.1 GBq (i.e., 30 mCi), thereby facilitating outpatient treatment. This approach is considered cost-effective, as it reduces hospital admission costs. In a retrospective study, Mc Cowen compared the efficacy of low-dose 1.1 GBq (i.e., 30 mCi) of I-131 with higher doses ranging from 3.0 to 3.7 GBq (i.e., 81–100 mCi) for remnant ablation. His findings indicated that higher doses did not result in superior efficacy in achieving successful ablation [51]. Several authors have suggested that when surgical resection is adequate, a dose of 0.925 GBq (i.e., 25 mCi) of iodine-131 is sufficient for effective thyroid remnant ablation [52,53]. In a randomized study involving 565 patients with DTC, Bal et al. assigned participants to eight groups based on the administered activity of radioactive iodine-131, ranging from 0.9 GBq to 1.85 GBq (i.e., 50 mCi), in 0.185 GBq (i.e., 5 mCi) increments. The authors reported that patients who received ≥ 0.9 GBq (i.e., ~24.3 mCi) of I-131 had a threefold higher likelihood of successful thyroid remnant ablation compared to those who received < 0.9 GBq (i.e., ~24.3 mCi). Any doses ranging from 0.9 GBq (i.e., ~24.3 mCi) to 1.85 GBq (i.e., 50 mCi) of I-131 appear to be adequate for effective remnant ablation [52]. In a follow-up study conducted several years later, the same authors demonstrated that a single dose of 0.925 GBq (i.e., 25 mCi) or 1.85 GBq (i.e., 50 mCi) was as effective for thyroid remnant ablation after total thyroidectomy as a 3.7 GBq (i.e., 100 mCi) dose [53]. These results were later supported by multiple studies reporting similar findings [54,55,56,57,58]. Four randomized studies comparing therapeutic doses of 30 mCi and 100 mCi demonstrated that 1.1 GBq (i.e., 30 mCi) was as effective as 3.7 GBq (i.e., 100 mCi) [54,55,56,57]. Similarly, Caglar et al. reported that 0.8 GBq (i.e., ~21.62 mCi) of I-131 effectively ablates remnant thyroid tissue in patients with low-risk well-differentiated thyroid cancer following total thyroidectomy. The success rate was comparable to that achieved with 3.7 GBq (i.e., 100 mCi) of I-131 [58].

Subsequently, two randomized multicenter prospective studies, HiLo in England and Estimabl 1 in France, were designed to evaluate whether low-dose iodine-131 activity (1.1 GBq, i.e., 30 mCi) is as effective as higher activity (3.7 GBq, i.e., 100 mCi) for thyroid remnant ablation. They also aimed to compare the efficacy of radioiodine ablation following preparation with either rhTSH or thyroid hormone withdrawal [59,60]. The HiLo study, conducted across 29 centers in England, included 438 patients classified as low to intermediate risk according to the TNM classification (T1-T3, N0, Nx, N1, M0), indicating possible spread to regional lymph nodes but no distant metastases [59]. The Estimabl 1 study, conducted across 24 centers in France, involved 752 low-risk patients classified as pT1 (≤1 cm)/N1/Nx, pT1 (>1–2 cm), any N, and pT2N0M0, based on the TNM classification [60]. In both studies, no statistically significant differences in the efficacy of radioiodine ablation were reported between the groups treated with 1.1 GBq (i.e., 30 mCi) and 3.7 GBq (i.e., 100 mCi) of I-131. In the HiLo study, the ablation rates were 85% for the group treated with 1.1 GBq (i.e., 30 mCi) and 87% for those treated with 3.7 GBq (i.e., 100 mCi) [59]. Similarly, in the Estimabl 1 study, the ablation rate was 92% for both 1.1 GBq (i.e., 30 mCi) and 3.7 GBq (i.e., 100 mCi) activity groups [60].

As noted in a review by Buscombe, further prospective, randomized studies with larger patient populations and longer follow-ups are needed to obtain statistically significant and reliable results [61].

### 3.4. Former Guidelines and Recommendations for DTC Management

The optimal treatment for differentiated thyroid carcinoma has been controversial for many decades. Given the low incidence, indolent course, and good prognosis of the disease, this is not surprising. The ongoing debate has prompted the development of standardized diagnostic and treatment strategies for managing DTC through a multidisciplinary approach. Today, many clinicians rely on established guidelines to determine the most effective treatment algorithms for DTC patients.

In general, the guidelines offer recommendations for the optimal treatment algorithm in patients with DTC. They do not encompass all appropriate approaches or exclude alternative methods. In addition, guidelines do not define a standard of care and do not guarantee specific clinical outcomes. However, treatment decisions should be based on the objective judgments of each patient. Clinical guidelines are designed to support—not replace—clinical decision-making in the management of patients, including diagnosis and treatment planning. Their basic role is to assist medical staff in providing safe and effective medical care. However, a significant limitation of current guidelines is the lack of large-scale, randomized clinical trials with long-term follow-up—studies that are inherently difficult to carry out.

Over the years, several guidelines have been developed and continuously refined in response to emerging research and new evidence. The first consensus on DTC management was established at the International Conference on the Management of Differentiated Thyroid Cancer and published in 1988 [62]. Almost a decade later, in 1996, the ATA released its initial guidelines for patients with thyroid nodules and DTC [63]. In the years that followed, ATA guidelines underwent multiple revisions and updates—in 2006 [64], 2009 [20], and most recently in 2016 [21]. In addition, several other publications have been issued by various medical societies around the world. Among the most widely cited are the guidelines published by the Society of Nuclear Medicine and Molecular Imaging (SNMMI) in 2012 [65] and the European Association of Nuclear Medicine (EANM) in 2008 [66]. In 2002, the British Thyroid Association (BTA) [67] and in 2006, the European Thyroid Association (ETA) [68] also published comprehensive guidelines, which have been periodically updated to reflect current best practices in the management of DTC [69,70,71].

The most recently published ATA guidelines faced widespread criticisms from the global thyroid cancer community. Notably, the EANM declined to endorse these guidelines, stating: “In spite of solid evidence on the clinical efficacy of nuclear medicine in both the diagnostic work-up of nodular thyroid disease and the care of DTC, the 2015 ATA guidelines marginalize the role of nuclear medicine in the care of nodular thyroid disease and DTC” [72].

Beyond the unsatisfactory response to the ATA 2015 guidelines, several controversial issues regarding the use of radioactive iodine-131 remain. Ongoing debates persist on several key issues, including the role of pre-treatment diagnostic whole-body scintigraphy with I-131 (I-131 WBS), the necessity of radioiodine ablation in low and intermediate-risk patients, the optimal radioiodine activity (i.e., dose), and the preferred method of administration. The main drawback of the existing guidelines is the lack of randomized clinical trials applied to large numbers of patients with long-term follow-up. To address these controversies, several recent guidelines on DTC management have been published.

## 4. Current Approaches for DTC Management

### 4.1. Current Guidelines and Recommendations

In 2022, four new guidelines addressing the management of DTC patients were published. The ETA released the “ETA Consensus Statement: What Are the Indications for Post-Surgical Radioiodine Therapy in Differentiated Thyroid Cancer?” [73]. In response to the ETA publication and its consensus recommendations, German surgeons and nuclear medicine experts released a position paper titled “Individualized Treatment of Differentiated Thyroid Cancer: The Value of Surgery in Combination with Radioiodine Imaging and Therapy—A German Position Paper from Surgery and Nuclear Medicine” [74], offering commentary and an alternative perspective on the ETA consensus. Soon after, the EANM and the SNMMI published joint guidelines “SNMMI Procedure Standard/EANM Practice Guideline for Nuclear Medicine Evaluation and Therapy of Differentiated Thyroid Cancer: Abbreviated Version” [75]. In addition, the National Institute for Health and Care Excellence (NICE) published “Thyroid Cancer: Assessment and Management” [76].

According to the latest recommendations [75,77], the use of radioactive iodine is categorized into three primary indications: (1) radioiodine ablation of normal thyroid remnants in low-risk patients to facilitate follow-up; (2) adjuvant therapy with the role of destroying suspected (unproven) tumor tissue in a group of patients with low- and intermediate risk (as determined by histopathological features) to reduce the risk of recurrence; (3) treatment of existing disease, focusing on the management of persistent or recurrent disease in patients with confirmed metastatic involvement. The goals of ablative radioiodine therapy include initial disease staging and facilitating postoperative patient monitoring. This is achieved through undetectable or minimally detectable serum thyroglobulin levels, absence of antithyroglobulin antibodies, and negative whole-body scintigraphy indicating the absence of residual neoplastic tissue. Beyond these purposes, adjuvant therapy serves additional roles: providing curative intent, prolongation disease-specific survival, extending progression-free survival, and reducing the risk of recurrence. The therapeutic roles in managing known disease include aiding in initial staging, facilitating postoperative monitoring, extending disease-specific survival, prolonging progression-free survival, and providing both curative and palliative benefits [75,77].

The decision to administer radioiodine therapy depends on the treatment goal and is guided by the estimated risk of mortality and the likelihood of persistent or recurrent disease [75]. The latest version of the TNM classification (8th version) estimates the risk of mortality from the underlying disease [18]. Unlike the risk of mortality, the risk of recurrence or persistent disease is much higher. The ATA 2015 guidelines modified the initial DTC risk stratification by incorporating three additional prognostic factors: lymph node involvement, mutation status, and the degree of vascular invasion in follicular thyroid carcinomas [20]. The recurrence risk differs among the different categories and accounts for 5–10%, 5–30%, or >20% for low, intermediate, and high-risk groups, respectively [21,73].

### 4.2. DTC Patient’s Risk Assessment

The currently recommended risk assessment for DTC patients includes three widely accepted risk categories: low, intermediate, and high risk [21].

#### 4.2.1. Patients with a Low Risk of Relapse/Resistant Disease

The low-risk group includes patients with PTC who have undergone complete resection of a macroscopically visible tumor with intra-thyroidal tumor size 2–4 cm, no vascular invasion, and favorable histological features (excluding aggressive variants such as columnar cell, tall cell, or hobnail carcinoma. These patients also show no invasion into surrounding loco-regional tissues and no radioiodine uptake outside the thyroid bed on the initial post-treatment I-131 whole-body scan if I-131 therapy was administered. Additionally, they have local or distant metastases and either no lymph node metastases or only small-volume metastases defined as clinical N0 or ≤5 pathologic N1 micrometastases, each measuring <0.2 cm in greatest dimension. The low-risk group also includes patients with intrathyroidal, encapsulated PTC follicular variant or intra-thyroid well-differentiated follicular carcinoma with capsular invasion or limited vascular invasion (<4 foci of vascular invasion). Finally, it includes patients with intrathyroidal papillary microcarcinoma (unifocal or multifocal) that harbors either wild-type BRAF or the BRAFV600E mutation, if known [21].

The impact of radioiodine therapy in patients with a low risk of recurrent disease remains controversial. The risk of mortality in this group of patients is very low (<1%), as is the risk of relapse/persistent disease (2–3%) [21,73]. In a study by Carhill et al., radioiodine ablation in low-risk patients showed no impact on overall survival [78]. A study of 1298 low-risk ATA patients, conducted by Schvartz et al. with a median follow-up of over 10 years, found that adjuvant therapy did not improve overall survival or disease-free survival [79]. In two prospective National Thyroid Cancer Treatment Cooperative Study Group (NTCTCSG) studies conducted five years apart, researchers analyzed patients with low-risk stages I and II disease, including those under 45 years of age without distant metastases and patients 45 years or older with tumors smaller than 4 cm, without extrathyroidal extension (ETE) or nodal metastases. Their results also showed that radioiodine ablation did not affect overall survival, survival from underlying disease, or disease-free survival [80,81]. Extensive systematic literature analyses have found no evidence that adjuvant radioiodine therapy reduces disease-specific mortality in low-risk patients. Additionally, data on its effect in reducing recurrence have been inconsistent [82,83,84]. Consequently, in a recent systematic review, Verburg et al. evaluated the impact of postoperative I-131 therapy on DTC recurrence and disease-specific survival. Their findings were mixed, with some studies showing a benefit and others not. Ultimately, they concluded that “at least until randomized prospective studies prove otherwise, the prescription of adjuvant I-131 treatment to all DTC patients with a primary tumor diameter exceeding 1 cm remains a reasonable option” [85]. According to the national guidelines of Great Britain (NICE), radioiodine therapy is recommended for patients with pT1a and pT1b tumors only if adverse features are present, such as aggressive histological subtypes or an R1 surgical resection margin (strong recommendation). In T2N0M0 patients, treatment with I-131 may have a beneficial effect and should be considered (conditional recommendation) [76]. The latest consensus from the ETA on radioiodine therapy in DTC suggests that ablation in low-risk patients should be based on individual assessment of risk factors. Radioiodine ablation does not influence disease outcomes and is not indicated for microcarcinomas without risk factors. However, low-risk patients with detectable postoperative serum thyroglobulin levels (≥2 ng/mL on LT4 therapy or >5–10 ng/mL after TSH stimulation) or a positive ultrasonographic finding of the neck have an increased risk of recurrence. While improved disease-free survival has not been clearly demonstrated in these patients, radioiodine ablation may be considered [73].

A recent systematic review and meta-analysis found that low-dose RAI is as effective as high-dose RAI in terms of ablation success and recurrence rates. These findings indicate that for patients with low- to intermediate-risk DTC, low-dose RAI therapy may be the better option due to its comparable effectiveness and lower risk of side effects [86].

Since the use of radioactive iodine in low-risk patients remains a subject of debate among clinicians, controlled randomized prospective studies are needed to determine its statistically significant impact on recurrence and mortality. Two prospective, randomized, non-inferiority trials investigating the role of radioiodine ablation in DTC are currently being conducted in Europe. The first, a British study titled “The IoN Study (Is Ablative Radio-iodine Necessary for Low-Risk Differentiated Thyroid Cancer Patients)”, compares outcomes in low-risk and selected intermediate-risk DTC patients treated with surgery alone versus those treated with surgery followed by RAI therapy [87]. The second, the French “Estimabl 2 study (Differentiated Thyroid Cancer: Is There a Need for Radioiodine Ablation in Low-Risk Patients)”, evaluates low-risk DTC patients who underwent total thyroidectomy (with or without neck dissection). Participants were randomized to receive either 1.1 GBq (i.e., 30 mCi) of I-131 following stimulation with recombinant human thyroid-stimulating hormone (rhTSH) or no RAI therapy. Initial results from a three-year follow-up indicated that RAI ablation did not provide a significant benefit in low-risk patients [88]. These findings were reinforced by a subsequent five-year follow-up publication, which further supported the conclusion that postoperative RAI therapy may not be necessary in this patient population [89].

However, several researchers have raised concerns regarding the validity of these conclusions [90,91,92]. Hence, in the Estimabl 2 study, RAI ablation was administered to patients with already undetectable thyroglobulin levels—contradicting the fundamental rationale for ablation, which is to achieve undetectable serum thyroglobulin as a surrogate marker of disease eradication. This raises ethical questions regarding the necessity of ablation in such cases [90]. In addition, a follow-up period of only three years is insufficient to fully assess recurrence rates in low-risk DTC patients, as disease recurrence can manifest beyond this timeframe. Furthermore, the effectiveness of administering 1.1 GBq (30 mCi) of I-131 for achieving remnant ablation remains uncertain in many patients [91]. Moreover, the study design has been criticized for methodological limitations. Notably, the response to RAI therapy is influenced by the absorbed radiation dose rather than the administered activity alone. Furthermore, differing thresholds for thyroglobulin levels were applied to the RAI and non-RAI groups, potentially biasing the assessment of treatment efficacy [92]. These limitations suggest caution in interpreting the Estimabl 2 results as definitive evidence against the utility of RAI ablation in low-risk DTC patients.

#### 4.2.2. Patients with Intermediate Risk of Relapse/Resistant Disease

The intermediate-risk group includes patients with one or more of the following characteristics: minimal ETE, defined as microscopic tumor invasion into the peri-thyroid soft tissues; tumors with aggressive histologic features, such as columnar cell carcinoma, tall cell carcinoma, hobnail carcinoma, or diffuse sclerosing carcinoma; PTC with vascular invasion; clinical N1 or more than 5 pathologic lymph node metastases (N1), all measuring less than 3 cm in greatest dimension; presence of iodine-avid foci in the neck outside the thyroid bed on the initial post-therapy I-131 WBS; or multifocal papillary microcarcinoma with microscopic tumor invasion into peri-thyroid soft tissues and BRAFV600E mutation, if known [21].

Data from this patient group indicate that I-131 therapy has the greatest impact on patients with adverse histological types (tall cell carcinoma, diffuse sclerosing carcinoma, insular carcinoma), nodal metastases, and older age [20]. Studies have reported that adjuvant therapy has a positive impact on overall survival, disease-specific survival, and disease-free survival in patients aged 45 years or older with nodal metastases (NTCTCSG Stage 3) [80]. In a study of 21,870 intermediate-risk patients (≤4 cm T1-3 N1 M0/x, >4 cm T3 N0 M0/x) with a median follow-up of six years, Ruel et al. demonstrated that adjuvant I-131 therapy significantly improved overall survival reducing mortality risk by 29%. A 36% reduction in mortality risk was associated with radioiodine therapy in patients younger than 45 [93]. Additionally, in an analysis of 8601 intermediate-risk patients with (T1/T2 N1 and T3 N0/1) from the SEER registry, of whom 67.6% received postoperative radioiodine, Zang found that radioiodine ablation improved overall survival but had no significant effect on disease-specific survival [94]. In a retrospective analysis of data on 8297 intermediate-risk PTC, 90.2% of whom underwent radioiodine ablation, Kim et al. reported contrasting findings. Namely, their results showed that radioiodine therapy did not significantly reduce loco-regional recurrence, even among patients with aggressive characteristics (BRAF positivity, with multifocal tumors, tumors > 1 cm, extra-thyroidal extension, and regional nodal metastases) [95]. Similarly, a systematic review conducted by Lamartine and colleagues on the effect of radioiodine therapy in intermediate-risk patients yielded differing results. According to an analysis of 13 publications, radioiodine therapy did not reduce recurrence in some cases, while 11 other studies confirmed its effectiveness [96]. In order to definitely determine the role of I-131 therapy in this group, large-scale prospective randomized studies are needed to provide more conclusive evidence.

#### 4.2.3. Patients with a High Risk of Relapse/Resistant Disease

The high-risk group is characterized by any of the following features: macroscopic tumor invasion into the peri-thyroid soft tissues (i.e., extensive ETE); incomplete tumor resection; pathologic N1 disease with any lymph node measuring ≥3 cm in the greatest dimension; distant metastases; elevated postoperative serum thyroglobulin suggestive of distant metastases; or follicular thyroid carcinoma with extensive vascular invasion, defined as more than 4 foci of vascular involvement [21].

The greatest impact of I-131 therapy was observed in patients with loco-regionally advanced disease and distant metastases. An analysis of the NTCTCSG registry, including 2936 DTC patients and conducted by Jonklaas et al., demonstrated that high-risk patients with advanced disease, regional and/or distant metastases, or stage 3 and stage 4 who received radioiodine therapy, experienced prolonged disease-specific survival and extended disease-free survival [80]. A few years later, Carhill et al. confirmed that radioiodine therapy positively impacted overall survival, but only in high-risk patients (stage 3 and stage 4), after analyzing the NTCTCSG registry of 4941 patients with a median follow-up of six years [78]. Similarly, Podnos et al., in an analysis of SEER registry data from 14,545 patients, found that radioiodine therapy significantly improved overall survival exclusively in high-risk PTC patients—those over 45 years old, with tumors larger than 2 cm, nodal metastases in the neck, and distant metastases [96]. Among patients with FTC and distant metastases, the application of radioiodine therapy doubled the overall survival of the patients, increasing it from 17.6% to 38.3% (*p* = 0.036) [97]. According to the NICE 2022 guidelines, radioiodine therapy is strongly recommended for patients with stage pT3, pT4, nodal metastases, poor prognostic histopathological features (including multifocality), or distant metastases (“strong recommendation”) [76]. A registry-based analysis on 11,832 stage IV patients found that those with PTC and FTC who received I-131 therapy had significantly prolonged 5- and 10-year survival compared to those who did not receive radioiodine treatment. The mortality rates of PTC patients treated with I-131 compared to those who did not receive the therapy were 11% vs. 22.7% after 5 years and 14% vs. 25.5% at 10 years. For FTC patients who received I-131, mortality was 29.2% at 5 years and 36.8% at 10 years, whereas in those who did not receive I-131, the rates were significantly higher—45.5% at 5 years and 51% at 10 years [98].

### 4.3. Radioiodine Treatment Strategies

Current recommendations propose two approaches for I-131 therapy in DTC treatment: a risk-based approach which integrates clinical and histopathological factors along with institutional (local) protocols, and a theranostic approach, which relies on diagnostic postoperative whole-body scintigraphy using I-131 or I-123 (I-123 WBS, I-131 WBS) [75].

#### 4.3.1. Risk Adapted Treatment Approach

This approach, known as the empiric approach, is commonly used in nuclear medicine centers. It is based on institutional protocols, clinical experience, resource availability, and patient-specific factors. Current clinical practice typically involves administering empiric I-131 activities ranging from 1.85 to 7.4 GBq (i.e., 50–200 mCi). According to this approach, the prescribed I-131 activity is guided by the therapeutic goal and tailored to the patient’s risk for recurrence or persistent disease. Suggested I-131 activities include the following: 1.11–1.85 GBq (i.e., 30–50 mCi) for remnant ablation in low-risk patients; 1.85–3.7 GBq (i.e., 50–100 mCi) for adjuvant treatment; 3.7–5.55 GBq (i.e., 100–150 mCi) for small-volume loco-regional disease; and 5.55–7.4 GBq (i.e., 150–200 mCi) for advanced loco-regional disease and/or small-volume distant metastatic disease. In patients with diffuse distant metastatic disease, higher I-131 activities ≥7.4 GBq (i.e., ≥200 mCi) are recommended, guided by dosimetric calculations to optimize therapeutic efficacy and minimize toxicity [75].

#### 4.3.2. Theranostic Approach

This approach includes the performance of diagnostic imaging for radioiodine therapy planning. Postoperative diagnostic WBS is recommended as an imaging tool to detect and localize radioiodine-avid metastases. Depending on the obtained imaging data, patient management may be adjusted by omitting radioiodine therapy, referring the patient to additional surgery, modifying the prescribed I-131 activity, or performing a dosimetric study for metastatic DTC. Disadvantages of the empiric approach versus dosimetric studies for the treatment of patients with locoregional and metastatic disease have been reported. Specifically, the empiric approach does not account for the minimum I-131 activity required to deliver a therapeutic effect or the maximum safe absorbed dose to avoid toxicity. As a result, this approach may lead to overtreatment, exposing the patient to unnecessary radiation or under-treatment, where insufficient initial I-131 activity compromises treatment effectiveness [75]. In contrast, the dosimetric approach determines the I-131 activity individually for each patient.

##### Dosimetry-Guided Approach

There are two dosimetric approaches that have been proposed for the individualized treatment of DTC. This method is primarily used to treat DTC patients with distant metastases, extensive lung metastases, pediatric DTC, and elderly DTC patients [21]. It is based on dosimetric studies and calculations and includes two methods: blood or bone marrow dosimetry and lesion dosimetry.

The first method, bone marrow dosimetry, prioritizes safety by focusing on blood as a surrogate for red bone marrow, the critical organ most susceptible to radiation-induced myelotoxicity. This method is more commonly used than lesion dosimetry and focuses on determining the highest safely administrable dose of radioactive iodine. It was first introduced by Benua et al., who defined the “Maximum Tolerated Dose” (MTD) as the maximum I-131 activity that can be administered without causing significant bone marrow toxicity [99]. Permanent bone marrow suppression does not occur when the absorbed dose to the blood remains below 2 Gy. This approach requires serial blood sampling to measure radiation counts and probe-based whole-body activity measurements over a monitoring period of at least four days following administration of a diagnostic dose of I-131. The EANM Dosimetry Committee has published guidelines for standardized dosimetry procedures aimed at ensuring that the administered activity of I-131 does not exceed a 2 Gy blood-absorbed dose [100]. This blood dosimetry-based method allows for the safe administration of therapeutic doses exceeding 5.55 GBq (i.e., 150 mCi) and, in select cases, up to 12.95–18.5 GBq (i.e., 350–500 mCi) or higher. Importantly, it also identifies patients who may not safely tolerate even standard therapeutic doses (<5.55 GBq, i.e., 150 mCi). Furthermore, in patients with iodine-avid pulmonary metastases, whole-body retention of I-131 at 48 h post-administration should not exceed 2.96 GBq (i.e., 80 mCi). If such lesions are not present, a retention threshold of up to 4.44 GBq (i.e., 120 mCi) is considered acceptable [75].

The second approach, lesion-based dosimetry, proposed by Maxon et al., aims to optimize treatment efficacy by targeting sufficient radiation-absorbed doses to the tumor sites while minimizing side effects on non-target tissues [101]. To calculate the radiation-absorbed dose to a specific lesion, it is necessary to determine both the iodine-131 uptake and clearance kinetics for each lesion, along with the lesion’s mass or its effective volume of distribution. Whole-body scintigraphy images are typically acquired at multiple time points—up to 96 h following tracer administration—to assess these parameters; however, in certain cases, imaging at later time points may be warranted. Quantification of I-131 activity within lesions requires defining regions of interest (ROIs) using gamma camera imaging modalities such as planar scintigraphy, single photon emission computed tomography (SPECT), or I-124 positron emission tomography and computed tomography (PET/CT). To enhance accuracy, attenuation and scatter corrections are recommended, utilizing either transmission scans or scatter-based imaging techniques. The calculation of the administered activity necessary to achieve a target absorbed dose typically involves modifications of the standard Medical Internal Radiation Dose (MIRD) formalism. The disadvantage of this method lies in uncertain dosimetric calculations, including different reports on absorbed dose thresholds to achieve optimal therapeutic outcomes. Thus, Maxon et al. [101] suggest a minimum absorbed dose of 300 Gy for thyroid remnants and 80 Gy for lymph node metastases in contrast to Flux [102] and Wierts [103], who proposed at least 49 Gy and 90 Gy for thyroid remnants, respectively.

Some centers combine both lesion and blood dosimetry methods, though accurately calculating the lesion volume for metastatic and remnant tissue remains one of the main challenges in this individualized treatment approach [104]. According to the ATA 2015 Guidelines, “no recommendation can be made regarding the superiority of one method of RAI administration over another (empiric high activity vs. blood and/or body dosimetry vs. lesion dosimetry)” [21]. In patients with diffuse lung metastases, dosimetry-guided treatment is recommended to identify those at risk of side effects and to determine the highest safe activity that maximizes tumor radiation. To minimize the risk of significant myelosuppression and radiation pneumonitis, whole-body retention of I-131 at 48 h should not exceed 2.96 GBq (i.e., 80 mCi) in patients with iodine-avid diffuse pulmonary metastases or 4.44 GBq (i.e., 120 mCi) if those lesions are absent [105].

To date, no conclusive published evidence demonstrates a definitive advantage of the dosimetric approach over the empiric method in treating locoregional or metastatic thyroid disease. Nevertheless, some studies have presented contrasting findings. Recent reports suggest that dosimetry-guided radioiodine therapy may offer clinical benefits over the standard approach [106,107]. Many researchers have suggested that a personalized dosimetric approach provides better patient survival and improves treatment effectiveness [108].

**Figure 1:**
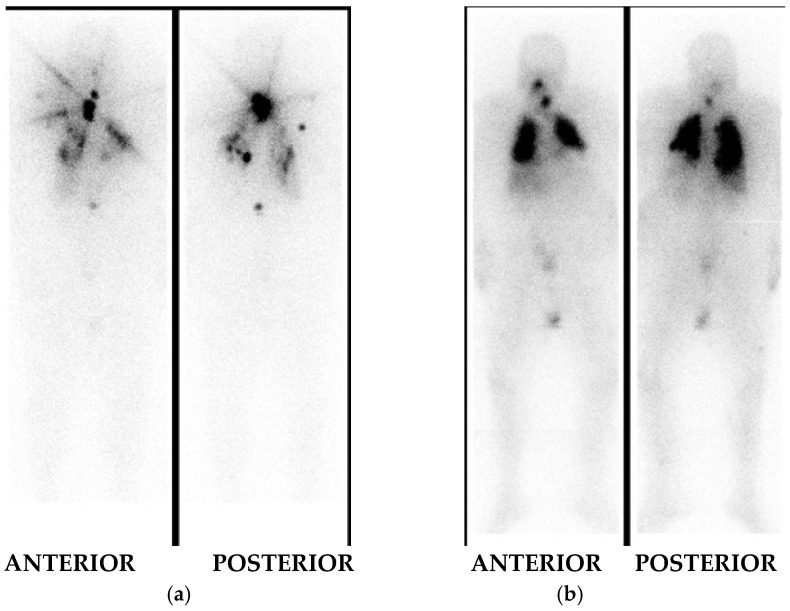
Post-treatment WBS planar images, anterior and posterior planes; (**a**) Post-treatment whole-body imaging following I-131 therapy with 1.2 GBq demonstrates radiotracer uptake in the thyroid bed consistent with residual tissue in the left thyroid lobe. Additional foci of increased activity in both lungs corresponds to pulmonary metastases, and one focus in the lumbar spine is consistent with bone metastasis; (**b**) Post-treatment whole-body imaging following I-131 therapy with 10.7 GBq shows a new focal area of uptake in the left cervical region, consistent with a metastatic lymph node. Diffuse uptake persists in both lungs, suggestive of widespread pulmonary metastases. The previously visualized lumbar spine lesion is no longer evident, indicating interval resolution of that metastatic focus.

Although dosimetric studies are complex and inconvenient for patients, they offer the advantage of personalized treatment for DTC patients with metastatic disease. An example of therapeutic efficacy using blood-based dosimetry is presented in Figure 1. Patient with an insular variant of DTC who had previously undergone left thyroid lobectomy received an initial dose of I-131 therapy at 1.2 GBq (~32.4 mCi). Post-treatment whole-body imaging revealed normal residual thyroid tissue in the left lobe, along with bilateral lung metastases and a metastatic lesion in the lumbar spine. Three months later, a second dose of I-131 therapy (10.7 GBq, ~289.2 mCi) was administered, based on dosimetric analysis and the calculated maximum tolerated activity. Subsequent whole-body scintigraphy revealed a newly detected metastatic lymph node in the left cervical region, along with persistent, diffuse pulmonary metastases in both lungs. The previously visualized lumbar spine metastasis was no longer evident, suggesting a complete therapeutic response at that site.

##### I-131 Whole Body Imaging

Radioiodine imaging in the postoperative setting for DTC patients includes both pre-treatment and post-treatment whole-body scanning. The use of diagnostic pre-treatment whole-body scintigraphy with radioactive iodine after total thyroidectomy remains controversial. While some authors support this method, others oppose it [109,110]. This imaging is used to assess the size of postoperative thyroid tissue remnants and to detect residual or previously unrecognized regional or distant metastases. Pre-treatment WBS is usually performed using a low activity of I-131, 37–185 MBq (i.e., 1–5 mCi), with low sensitivity for detecting occult disease [111]. Higher I-131 activity can enhance lesion detection and diagnostic sensitivity but may also increase the risk of thyroid stunning [112,113,114]. By reducing the uptake of a therapeutic dose of I-131, stunning can impair the effectiveness of radioiodine treatment [115]. Hence, radioactive iodine-123 (I-123) is recommended as an alternative to I-131 because it does not cause the “stunning phenomenon”. Although I-123 offers a lower radiation-absorbed dose, low background activity, and better image quality, its availability is limited—due to higher costs [116,117].

While most of the nuclear medicine centers in the US continue to apply the pre-treatment WBS, many institutions, especially in Europe, have abandoned this practice in recent years. This is primarily due to recent guideline recommendations from several guidelines for the management of DTC, which do not support the routine use of pre-treatment I-131 WBS [66,70,73,118]. The SNMMI procedure standard/EANM practice guidelines recommend the use of pre-treatment I-131 WBS for planning radioiodine therapy in intermediate and high-risk DTC patients. In addition, the guidelines note that, whenever possible, this diagnostic procedure should be performed using integrated multimodal imaging, specifically SPECT/CT [75].

Post-treatment WBS plays a significant role in the initial staging of radioiodine-avid DTC and in the identification of non-radioiodine-avid tumors. Integrated multimodal imaging, such as SPECT/CT, enhances planar whole-body imaging. In the post-treatment setting, it provides more accurate initial TNM staging and better risk stratification for DTC patients [75]. Post-treatment I-131 imaging is mandatory for all DTC patients who are receiving radioiodine therapy.

### 4.4. Preparation Methods for Radioiodine Therapy

Recommendations for patient preparation before radioiodine therapy include adherence to a low-iodine diet and ensuring adequate TSH stimulation, both of which are essential for maximizing I-131 uptake by thyroid remnants and metastatic thyroid tissue.

#### 4.4.1. Low-Iodine Diet

A low-iodine diet before I-131 treatment is essential to eliminate excess iodine from food and medications, as excessive iodine can reduce the effectiveness of radioiodine therapy. Dietary iodine should be restricted for 1–2 weeks before ablation [119,120]. Reducing daily iodine intake to less than 50 μg can double iodine uptake, leading to more effective ablation [121].

Patients should avoid iodized salt, dairy products, eggs, seafood, and iodine-rich contrast media. Additionally, iodine-containing medications such as amiodarone, expectorants, and topical antiseptics should be discontinued based on medical guidance [119,120]. Some authors reported that patients following a low-iodine diet experience significantly improved radioiodine ablation efficacy compared to those in control groups (65% vs. 48%, *p* < 0.001) [122]. A recent study found that in areas with sufficient iodine intake, a low-iodine diet for just 4 days is enough to effectively deplete iodine levels in patients preparing for I-131 therapy [123].

#### 4.4.2. TSH Stimulation

The effectiveness of the radioiodine ablation depends on achieving an optimal hypothyroid state and ensuring adequate TSH stimulation. A TSH level of ≥30 mU/L is considered ideal for enhancing Sodium Iodide Symporter (NIS) expression and maximizing radioiodine uptake. This can be achieved naturally by withholding thyroid hormone therapy for 4–6 weeks postoperatively. Patients on thyroid hormone replacement therapy, such as levothyroxine (LT4), must discontinue treatment for the same duration prior to radioiodine ablation. An alternative approach involves substituting LT4 with liothyronine (LT3) and then discontinuing LT3 for two weeks before ablation to induce the hypothyroid state [75].

Another option is maintaining thyroid hormone replacement while rhTSH (Thyrogen^®^). This method involves two intramuscular injections of 0.9 mg rhTSH given 24 h apart before radioiodine treatment. It was approved by the U.S. Food and Drug Administration (FDA) in 1998 and by the European Medicines (EMA) in 2001 [124,125]. This approach raises TSH levels without inducing hypothyroidism and is generally well tolerated. Unlike thyroid hormone withdrawal, which often results in debilitating hypothyroid symptoms and limits the patient’s ability to work or engage in daily activities, preparation with rhTSH offers a better short-term quality of life. Additionally, rhTSH is especially recommended for patients with conditions that make prolonged hypothyroidism risky or poorly tolerated. These include cardiac disease, the postpartum period, hypopituitarism, psychiatric disorders, and cases in which patients cannot tolerate prolonged hypothyroidism [126,127].

According to the current ATA guidelines, rhTSH preparation is recommended for remnant ablation or adjuvant therapy in low- or intermediate-risk patients without extensive lymph node involvement (T1-T3, N0/Nx/N1a, M0). However, the FDA has not approved the use of rhTSH for patients with metastatic disease (20). Studies suggest no significant difference in ablation success rates between patients prepared with rhTSH and those undergoing hormone withdrawal [128]. Despite its advantages, rhTSH remains less widely available due to its high cost.

In a recent study, Lima et al. demonstrated that the type of preparation for RAI therapy did not significantly impact the outcomes in patients with metastatic DTC undergoing RAI therapy, whether prepared with rhTSH or thyroid hormone withdrawal (THW). Furthermore, no statistically significant difference in PFS was observed between the two preparation methods [129]. Another study reported results for preparation with both THW and hTSH stimulation in PTC with distant metastases: The 10-year disease-specific survival were 62.2% and 73.3%, respectively. Moreover, patients with RAI-avid metastases and rhTSH-based preparation demonstrated significantly better PFS compared to those in other groups (*p* = 0.025). It seems that rhTSH stimulation for RAI therapy preparation is not inferior to THW in terms of long-term survival outcomes for patients with papillary thyroid carcinoma and distant metastasis [130].

Recently, two innovative studies have explored the use of rhTSH as an alternative to thyroid hormone withdrawal for preparing patients with DTC for RAI therapy [131,132]. A 2022 Phase I/II trial evaluated a novel rhTSH formulation, ZGrhTSH, in Chinese patients with DTC. The results indicated that ZGrhTSH effectively stimulated RAI uptake and increased serum thyroglobulin levels. Importantly, patients reported improved quality of life during the ZGrhTSH phase compared to the period of levothyroxine (L-T₄) withdrawal, suggesting that ZGrhTSH may serve as a well-tolerated and effective alternative for RAI preparation treatment [131]. Another study, a Phase III randomized clinical trial published in 2024, evaluated the safety and efficacy of SNA001, a prefilled rhTSH injection, in comparison to conventional THW in intermediate-risk DTC patients. Conducted with 307 participants, the study found that SNA001 was non-inferior to THW in achieving successful RAI ablation. Moreover, the SNA001 group experienced fewer adverse effects, with safety and quality of life treatment [132].

### 4.5. Dynamic Risk Stratification

Initial risk stratification for DTC is performed postoperatively as a static, one-time assessment that traditionally remains unchanged throughout a patient’s lifetime. However, as the disease progresses, the recurrence risk in DTC patients evolves based on their response to the initial treatment [22]. Therefore, DTC patients need to be risk re-stratified during the follow-up. This “ongoing” or “dynamic” risk stratification presents a real-time risk evaluation that can be performed at any point during follow-up and allows for subsequent treatment adjustments. Through dynamic risk stratification, patients may shift between categories, moving to either a lower or higher risk category. For example, many DTC patients initially classified in the intermediate or high-risk categories may be reclassified to the low-risk category after an excellent response to treatment. As a result, a less aggressive treatment approach should be considered for these patients without increasing the risk of recurrence [22,23].

Dynamic risk stratification has been studied only in patients who have completed initial treatment, including total thyroidectomy and radioiodine ablation. It has not been evaluated in patients following partial initial treatment (after unilateral lobectomy only or after total thyroidectomy without radioiodine ablation) [133].

According to current recommendations, patients should undergo re-stratification within the first 1–2 years after initial therapy, based on their response to treatment. Treatment outcomes are categorized into four distinct response groups. An *excellent response* is defined as the complete absence of clinical, biochemical, and structural evidence of disease, including negative imaging and Tg levels (suppressed, Tg < 0.2 ng/mL or stimulated Tg < 1 ng/mL). Patients within this category show a low likelihood of recurrence, approximately 1–4%. In contrast, individuals with intermediate- or high-risk have higher recurrence rates, estimated at 36–45% and 68–70%, respectively. Therefore, complete remission achieved in higher-risk patients represents a great clinical benefit. Subsequent management for such patients typically involves less frequent monitoring and a reduction in the intensity of thyroid-stimulating hormone (TSH) suppression therapy [23,75]. 

**Figure 2:**
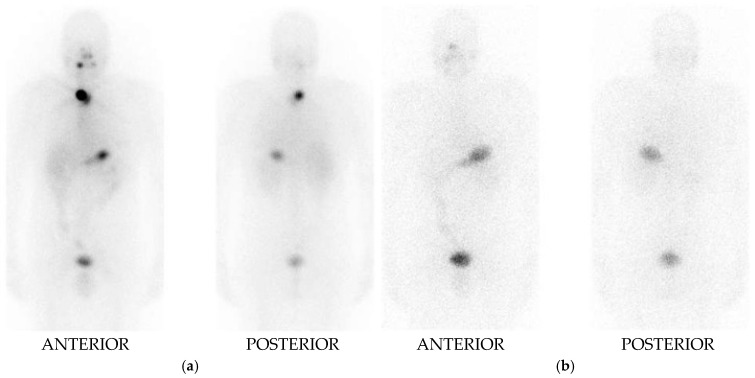
(**a**) Post-treatment WBS planar images, anterior and posterior planes, demonstrate increased uptake in the thyroid bed, consistent with residual postoperative thyroid tissue; (**b**) Diagnostic I-131 WBS planar images, anterior and posterior planes, performed one year later, show no abnormal uptake in the thyroid bed or elsewhere.

An example of an excellent therapeutic response—defined by the absence of clinical, biochemical, and structural evidence of disease—is illustrated in Figure 2. The patient, diagnosed with classic papillary thyroid carcinoma (T1bN1aM0, Stage I), underwent total thyroidectomy with central neck dissection, followed by adjuvant I-131 radioiodine therapy at a dose of 5.55 GBq. Pre-therapeutic laboratory evaluation demonstrated a TSH level >100 mIU/mL, serum thyroglobulin (Tg, measured by ECLIA) of 8.14 ng/mL, and undetectable anti-thyroglobulin antibodies (TgAb). At one-year follow-up, the therapeutic response was assessed: 24-hour radioiodine uptake measured 0.4%, TSH remained >100 mIU/mL, Tg decreased to 0.6 ng/mL, and TgAb remained undetectable. Initial post-treatment whole-body imaging showed uptake consistent with residual thyroid tissue. However, a diagnostic I-131 whole-body imaging performed one year later showed no iodine uptake in the thyroid bed, indicating complete remnant ablation and confirming an excellent therapeutic response.

A *biochemical incomplete response* is characterized by abnormal Tg levels (suppressed Tg > 1 ng/mL or stimulated Tg > 10 ng/mL) or increasing anti-thyroglobulin antibody (TgAb) titers in the absence of identifiable structural disease, i.e., negative imaging. Long-term follow-up data indicate that approximately 60% of these patients will not develop structural evidence of disease. Around 20% will continue to exhibit elevated Tg or TgAb levels without anatomical abnormalities, while the remaining 20% will progress to structural disease within 5–10 years. Patients showing rising Tg or TgAb levels warrant further evaluation through additional imaging studies and may require further radioiodine therapy [23,75].

Persistent or newly detected loco-regional disease or distant metastases define an *incomplete structural response*. Management of patients in this category depends on several factors, including the location of metastases (regional versus distant) and the radioiodine avidity of the lesions. These patients typically require additional therapeutic interventions, which should be guided by a multidisciplinary team [23,75].

An *indeterminate response* is characterized by nonspecific biochemical findings—such as suppressed Tg levels of 0.2–1 ng/mL, stimulated Tg 1–10 ng/mL, or stable/decreasing TgAb levels—or structural findings that cannot be definitively classified as benign or malignant (e.g., indeterminate imaging results and/or faint uptake on I-131/I-123 WBS). In most patients (80–90%) with indeterminate responses, the nonspecific biochemical status remains stable or shows resolution over time with LT4 therapy [23,75].

A recent systematic review by Bellini et al. reassessed the prognostic significance and clinical features of patients with differentiated thyroid carcinoma (DTC) who exhibit an indeterminate response. The review reported a lower recurrence risk, ranging from 1.3% to 14% [134], compared to the 15–20% estimate cited in the 2015 ATA guidelines [21]. This new evidence supports favoring active surveillance over the immediate initiation of additional therapy or diagnostic examinations, as previously advocated [135]. Furthermore, Bellini et al. investigated the relationship between thyroglobulin (Tg) levels and disease recurrence. Within this context, certain studies in the review identified stimulated Tg thresholds of 3.1 ng/mL and 3.3 ng/mL, along with a suppressed Tg cut-off of 0.35 ng/mL, as potentially optimal predictors of disease recurrence. Elevated stimulated Tg levels in the 1–10 ng/mL range may serve as an early indicator of persistent or recurrent disease in this patient cohort, potentially preceding radiologically detectable structural abnormalities. Consequently, closer follow-up, with more frequent imaging and serial Tg measurements, may be warranted to enable early detection of structural disease recurrence and to track biochemical trends over time [134].

### 4.6. Molecular Profiling of Thyroid Cancer

Molecular theranostics is a big step forward in understanding and treating DTC, making it possible to offer more personalized care. Emerging cancer classification systems place greater emphasis on genetic alterations and their biological impact, offering insights that go beyond traditional histopathological analysis. By combining molecular pathology with genomic profiling and advanced molecular imaging, clinicians can achieve a more nuanced understanding of tumor behavior [136].

In DTC, key mutations such as BRAFV600E, RAS, and TERT promoter mutations play a pivotal role in defining tumor differentiation, aggressiveness, and prognosis. The BRAFV600E mutation, commonly found in papillary thyroid carcinoma, is associated with more aggressive tumor features, higher recurrence rates, and reduced radioiodine avidity. RAS mutations, more commonly found in follicular thyroid cancer and the follicular variant of papillary thyroid carcinoma, are typically associated with well-differentiated tumors but may also indicate disease progression in certain contexts. TERT promoter mutations, especially when coexisting with BRAF or RAS mutations, are associated with higher-risk disease, poorer outcomes, and increased mortality [137,138,139,140,141,142,143].

In 2014, The Cancer Genome Atlas (TCGA) Research Network published a comprehensive molecular analysis of 496 PTC cases, revealing two key genetic drivers—BRAFV600E and RAS mutations. These mutations are associated with distinct tumor signaling pathways and behaviors. In their research, scientists developed a thyroid differentiation score (TDS) to categorize tumors based on how aggressive they are and how they might respond to treatment. This scoring method set the stage for more tailored therapies, which are now being tested in clinical trials [144,145].

Most recently, McGordon et al. analyzed mRNA expression data from 59 papillary thyroid cancer samples with matched normal tissues from TCGA. They examined how the expression of thyroid-specific metabolic genes changes in cancer, focusing on differences by mutation type: BRAFV600E, RAS, other mutations, or no known mutations. They found that genes important for iodine uptake and metabolism were significantly downregulated, particularly in cancers with the BRAFV600E mutation. In contrast, RAS-mutated tumors retained more of this function. The study suggests that assessing each tumor’s Thyroid Differentiation Profile (TDP) could help predict how well it might respond to RAI therapy. This personalized approach could guide decisions on the extent of surgery and the use of postoperative RAI treatment [146].

## 5. Future Directions

Looking ahead, the integration of molecular theranostics is expected to play a pivotal role in shaping the future of I-131 therapy for DTC. Advances in molecular imaging and targeted therapies are enabling clinicians to more accurately identify which patients will benefit from RAI, thereby reducing unnecessary treatment and reducing associated side effects. Molecular profiling is increasingly influencing treatment decisions, guiding the extent of surgery and the need for postoperative RAI. Incorporating molecular markers into clinical practice allows for a more personalized approach- informing on not only RAI use but also follow-up frequency care and the potential need for targeted therapies. As our understanding of thyroid cancer genomics deepens, molecular classification is expected to become a standard element in individualized care, enabling clinicians to tailor therapy based on the biological behavior of each tumor.

Future research should aim to refine risk stratification, particularly for low-risk patients, and investigate optimal RAI dosing strategies across different molecular subtypes. The clinical value of remnant ablation and the long-term impact of RAI therapy remain areas of ongoing debate and require further study. Additionally, active clinical trials exploring new radiopharmaceuticals, radioimmunotherapy, and targeted agents will be crucial in determining how best to integrate these innovations into routine clinical workflows.

The aim is to move away from a one-size-fits-all treatment approach and towards a more personalized method that takes into account the biological and genetic makeup of each tumor. As research progresses, the approach to RAI therapy will also continue to evolve, ensuring it stays a safe, effective, and tailored treatment for those with differentiated thyroid cancer.

## 6. Conclusions

The role of radioactive iodine in the management of differentiated thyroid cancer has undergone a significant transformation—from routine, broad use to a more selective and risk-adapted application. This evolution reflects advances in molecular profiling and risk assessment along with the introduction of updated guidelines such as the 2015 ATA Guidelines, the 2022 ETA Consensus Statement, and joint recommendations from SNMMI and EANM. These guidelines and recommendations point to a more personalized treatment approach, focusing on management benefits and reducing or omitting unnecessary procedures. Molecular markers and genetic information are important for patient's treatment decisions, including decisions on the extent of initial surgery, the necessity of postoperative radioiodine treatment, and how long patients should be monitored.

However, several questions remain controversial, especially about how to manage low and intermediate-risk patients, I-131 activity and management of radioiodine refractory patients. These issues highlight the need for ongoing research and well-designed clinical trials. In addition, the introduction of new radiopharmaceuticals, targeted therapies, and radioimmunotherapy are needed.

Today, radioiodine therapy for DTC is shifting to a more personalized approach that incorporates the tumor’s genetic and molecular profile. This ensures that RAI remains a safe and effective treatment option for thyroid cancer patients.

## Figures and Tables

**Table 1:** Evolution of the AJCC/TNM Classification System: Comparison of the 6th, 7th, and 8th Editions.

TNM	6th Edition	7th Edition	8th Edition *
T			
Tx	T cannot be assessed	T cannot be assessed	T cannot be assessed
T0	No evidence of T	No evidence of T	No evidence of T
T1	Tumor ≤ 2 cm limited to the thyroid	Tumors of ≤2 cm are limited to the thyroid	Tumors of ≤2 cm are limited to the thyroid
T1a	-	Tumor size ≤ 1 cm, limited to the thyroid	Tumor ≤ 1 cm, limited to the thyroid
T1b	-	Tumor size > 1 cm ≤ 2 cm, limited to the thyroid	Tumor > 1 cm ≤ 2 cm, limited to the thyroid
T2	Tumor > 2 < 4 cm limited to the thyroid	Tumor size ≤ 4 cm limited to the thyroid	Tumor size > 2 cm but ≤4 cm, limited to the thyroid.
T3	Tumor > 4 cm limited to the thyroid or any T with minimal ETE (extension to sternohyoid muscle or perithyroid soft tissues)	Tumor size > 4 cm limited to the thyroid or any T with minimal ETE (e.g., extension to sternohyoid muscle or perithyroid soft tissue)	Tumor size > 4 cm, limited to the thyroid or any T with gross ETE invading only strap muscles
T3a	-	-	Tumor > 4 cm, limited to the thyroid
T3b	-	-	Any T with gross ETE invading only strap muscles (e.g., extension to sternothyroid, sternohyoid, thyrohyoid, or omohyoid muscles)
T4a	Any T extending beyond the thyroid capsule to invade subcutaneous soft tissues, larynx, trachea, esophagus, or recurrent laryngeal nerve	Any T extending beyond the thyroid capsule to invade subcutaneous soft tissues, larynx, trachea, esophagus, or recurrent laryngeal nerve—moderately advanced disease	Any T with gross ETE invading subcutaneous soft tissues, larynx, trachea, esophagus, or recurrent laryngeal nerve
T4b	Tumor invades prevertebral fascia or encases carotid artery or mediastinal vessels	Tumor invades prevertebral fascia or encases the carotid artery or mediastinal vessels-very advanced disease	Any T with gross ETE invading prevertebral fascia or encasing the carotid artery or mediastinal vessels
N			
Nx	RLN cannot be assessed	RLN cannot be assessed	RLN cannot be assessed
N0	No RLN metastasis	No RLN metastasis	No RLN metastasisOne/more cytological or histologically confirmed benign LN
N0a	-	-	No radiologic or clinical evidence of locoregional metastasis
N1	RLN metastasis	RLN metastasis	RLN metastasis
N1a	Metastasis to level VI (pretracheal, paratracheal, and prelaryngeal/Delphian LN)	Metastasis to level VI (pretracheal, paratracheal, and prelaryngeal/Delphian LN)	Metastases to level VI or VII (pretracheal, paratracheal, or prelaryngeal/Delphian, or upper mediastinal) lymph nodes. This can be unilateral or bilateral disease
N1b	Metastasis to unilateral, bilateral, or contralateral cervical or superior mediastinal LN	Metastasis to unilateral, bilateral, or contralateral cervical or superior mediastinal LN (level VII)	Metastasis to unilateral, bilateral, or contralateral lateral neck lymph nodes (levels I, II, III, IV or V) or retropharyngeal lymph nodes
M			
Mx	Distant metastasis cannot be assessed	-	-
M0	No distant metastasis	No distant metastasis is found	No distant metastasis is found
M1	Distant metastasis is present	Distant metastasis is present	Distant metastasis is present

T-primary tumor, N-regional lymph node (RLN), lymph node (LN), M-distant metastasis, ETE-extrathyroidal extension; * All categories must be divided into subgroups: (s) solitary tumor (m) multifocal tumor (the largest tumor determines classification).

**Table 2:** Evolving AJCC/TNM Staging System—Comparison of 6th, 7th and 8th edition.

STAGING GROUPS											
6th Edition				7th Edition				8th Edition			
<45 years				<45 years				<55 years			
S	T	N	M	S	T	N	M	S	T	N	M
I	Any T	Any N	M0	I	Any T	Any N	M0	I	Any T	Any N	M0
II	Any T	Any N	M1	II	Any T	Any N	M1	II	Any T	Any N	M1
III	-	-	-	III	-	-	-	III	-	-	-
IVa	-	-	-	IVa			-	IVa	-	-	-
IVb	-	-	-	IVb	-	-	-	IVb	-	-	-
IVc	-	-	-	IVc	-	-	-	IVc	-	-	-
≥45 years				≥45 years				≥55 years			
S	T	N	M	S	T	N	M	S	T	N	M
I	T1	N0	M0	I	T1a, T1b	N0	M0	I	T1a, T1b, T2	N0	M0
II	T2	N0	M0	II	T2	N0	M0	II	T1, T2 T3	N1 N0, N1	M0 M0
III	T3 T1, T2, T3	N0 N1a	M0 M0	III	T3 T1, T2, T3	N0 N1a	M0 M0	III	T4a	Any N	M0
IVa	T1, T2, T3 T4a	N1b N0, N1a, N1b	M0 M0	IVa	T1, T2, T3 T4a	N1b N0, N1	M0 M0	IVa	T4b	Any T	M0
IVb	T4b	Any N	M0	IVb	T4b	Any N	M0	IVb	Any T	Any N	M1
IVc	Any T	Any N	M1	IVc	Any T	Any N	M1	IVc	-	-	-
